# Genome wide association analysis of cold tolerance at germination in temperate *japonica* rice (*Oryza sativa* L.) varieties

**DOI:** 10.1371/journal.pone.0183416

**Published:** 2017-08-17

**Authors:** Ester Sales, Juan Viruel, Concha Domingo, Luis Marqués

**Affiliations:** 1 Departamento Ciencias Agrarias y del Medio Natural, Instituto Universitario de Investigación en Ciencias Ambientales, Universidad de Zaragoza, Huesca, Spain; 2 Royal Botanic Gardens, Kew, Richmond, Surrey, United Kingdom; 3 Centro de Genómica, Instituto Valenciano de Investigaciones Agrarias, Moncada, Valencia, Spain; 4 Cooperativa de Productores de Semillas de Arroz, Sueca, Valencia, Spain; Western Australia Department of Agriculture and Food, AUSTRALIA

## Abstract

A pool of 200 traditional, landraces and modern elite and old cultivars of rice, mainly *japonica* varieties adapted to temperate regions, have been used to perform a genome wide association study to detect chromosome regions associated to low temperature germination (LTG) regulation using a panel of 1672 SNP markers. Phenotyping was performed by determining growth rates when seeds were germinated at 25° and 15°C in order to separate the germination vigorousness from cold tolerance effects. As expected, the ability to produce viable seedlings varied widely among rice cultivars and also depended greatly on temperature. Furthermore, we observed a differential response during seed germination and in coleoptile elongation. Faster development at 15°C was observed in seeds from varieties traditionally used as cold tolerant parents by breeders, along with other potentially useful cultivars, mainly of Italian origin. When phenotypic data were combined with the panel of SNPs for *japonica* rice cultivars, significant associations were detected for 31 markers: 7 were related to growth rate at 25°C and 24 to growth rates at 15°. Among the latter, some chromosome regions were associated to LTG while others were related to coleoptile elongation. Individual effects of the associated markers were low, but by combining favourable alleles in a linear regression model we estimated that 27 loci significantly explained the observed phenotypic variation. From these, a core panel of 13 markers was selected and, furthermore, two wide regions of chromosomes 3 and 6 were consistently associated to rice LTG. Varieties with higher numbers of favourable alleles for the panels of associated markers significantly correlated with increased phenotypic values at both temperatures, thus corroborating the utility of the tagged markers for marker assisted selection (MAS) when breeding *japonica* rice for LTG.

## Introduction

Quantitative trait loci (QTL) mapping and genome wide association studies (GWAS) have been used with genomic data, mostly single nucleotide polymorphisms (SNP), to dissect the genetic architecture of complex traits. While QTL mapping is generally based on biparental populations, GWAS identifies QTL based on the historic recombination in a panel of diverse germplasm, via the presence of linkage disequilibrium between SNPs and QTLs. The impact of these strategies in practical breeding programs, however, has been smaller than initially envisaged, mainly because the detected QTLs generally explain only a little portion of the phenotypic variance. Most traits are affected by large numbers of small-effect genes, and then negative QTL x QTL, QTL x genetic background or QTL x environment interactions may reduce the efficiency of marker assisted selection (MAS). Epistasis is also a factor accounting for this fail in the genetic gain, but conversely MAS can also be the only way of selecting favourable epistatic combinations [1]. More recently, whole genome prediction or genomic selection (GS) has been introduced as a tool to increase the rate of genetic progress achieved in plant breeding. GS combines dense molecular markers and phenotypes into linear regression models to obtain accurate predictions of genetic values that allow MAS of the most adequate parents. Although the aims of GWAS and GS are different, both approaches share common features, since they are based on a population for which phenotypic and genotypic data are available, and its predictions about associated markers and genotypes must be validated in advanced populations to test their applicability in plant breeding programs. To exploit GWAS results in GS, Zhang et al. [2] proposed, using data for 11 traits in rice, the adoption of a Best Linear Unbiased Prediction (BLUP) model including prior knowledge about the genetic regulation of the character or QTL regions detected in previous GWAS, which could improve the accuracy of GS in traits with low heritability. Furthermore, GWAS has been widely employed to dissect the genetic regulation of different traits that are interesting for rice genetic improvement [3–8], while Begum et al. [9] used for GWAS a population of adapted lines to accelerate the application of the identified QTLs to rice breeding, combining GWAS and GS in the same population. The accuracy of prediction based on GWAS/GS models has been recently corroborated by Spindel et al. [10].

One interesting trait for rice breeding in temperate regions is cold tolerance, since rice cultivation in these regions deals with low temperature stress as a main yield and flowering limitation [11]. Temperatures below 15°C usually induce low germination rates, yellowing or withering, reduced tillering, and delayed heading or sterility, which significantly reduce yield [12–14]. Specifically, germination ability at sub-optimal temperatures (Low Temperature Germination, LTG) is an important goal, since cold causes the retardation of development, while a vigorous growth during the seedling stage is crucial for an adequate plant establishment. Breeding rice for LTG is complicated as there are many factors limiting selection effectiveness. In a first place, the response of rice plants to cold depends on the developmental stage, then rice cold tolerance at one developmental stage is not necessarily correlated with the tolerance at other growth stages [15]. In a second place, LTG is hampered by its quantitative inheritance, since it is controlled by multiple genes distributed widely over the rice genome [16] with a significant environmental influence on its regulation. Third, there are epistatic interactions with pronounced non-random associations among many alleles at unlinked loci [17].

A wide range of intraspecific variation for LTG in rice has been reported, being genotypes belonging to subspecies *japonica* usually more cold tolerant than those from the *indica* group, probably as a result of the process of adaptation of *japonica* rice to temperate areas from its tropical region of origin [18]. Some *japonica* varieties are commonly used to improve cold tolerance of *indica* cultivars but, because of the generally low level of genetic diversity within the *japonica* gene pool, further improvement of elite *japonica* cultivars remains a huge breeding challenge [17].

Considerable efforts have been hence made to dissect the genetic regulation of cold tolerance using molecular markers and QTL mapping. As a consequence, many QTLs controlling LTG have been detected throughout the rice genome [16, 19–22] as well as QTLs related to cold tolerance at seedling stage [17, 18, 23–26]. More recently, a GWAS by Pan et al. [27] detected 22 QTLs associated to cold tolerance at germination in rice genome, with both positive and negative effects, using SSR markers and a panel of 174 Chinese accessions. However, the diversity in number and location of QTLs related to LTG in rice can also be explained by the different stress treatments (time, temperatures and duration) and indicators to describe cold tolerance (survival of seeds, germination rate, leaf roll and yellowing, etc.) that have been used by different authors [28]. Cruz and Milach [29] found that variables as reduction in coleoptile growth and percentage of seeds with coleoptile superior to 5 mm were more adequate to identify cold tolerant genotypes. Due to the presence of vigour differences among genotypes, plant performance at a low temperature is not exclusively dependent on the genotype cold tolerance. For this reason, some authors emphasize the importance of evaluating the performance under more than one temperature, enabling the separation of vigour from cold tolerance effects. In this work, the percentage of coleoptile length reduction due to low temperatures was the characteristic enabling the precise identification of genotypes previously known as cold tolerant or susceptible.

Among the *japonica* gene pool, the European varieties Arroz da Terra and Italica Livorno have been employed as cold tolerant parents in breeding programs [30, 31], and some advanced populations derived from these crosses have been used to map QTLs related to LTG [24]. The favourable qLTG3-1 allele of Italica Livorno detected in the chromosome 3 [19] has been cloned [32] and also detected in the cultivar Maratelli, which possesses other positive allele for LTG in chromosome 11, qLTG11-1 [33]. Using a population of recombinant inbred lines (RILs) derived from the Russian cultivar USSR5, Li et al. [34] identified three QTLs associated to LTG in chromosomes 7, 9 and 12, and fine mapped the locus showing the largest effect, qLTG9. Two seedling cold tolerance related QTLs, qCTS4 and qCTS12, have been also mapped in the chromosomes 4 and 12 of the American variety M-202 [11, 35]. Despite the availability of potentially useful donors and linked markers for the genetic improvement of LTG and CTS in rice cultivars, results of these studies have not been directly applicable to MAS owing to possible epistasis and gene × environment interactions associated with the identified QTLs [17]. Breeding is also limited by the presence of many undesirable characteristics in the cold tolerant donors.

In this context, we present here a GWAS performed in a diverse collection of 200 rice cultivars adapted to temperate regions, from a pool of modern elite and old cultivars, as well as traditional landraces, which are available for rice breeders in this zone [36]. The cultivars were genotyped using a panel of 2697 SNPs with wide coverage throughout the rice genome, and population structure and phylogenetic analyses showed a strong substructure, predominantly based on its grain type and origin (Spain, Italy, Japan, USA, Australia or Philippines, among others). The collection encompasses some cold tolerant cultivars, as Arroz da Terra, Italica Livorno, Kasalath, Koshihikari, M-202 or Maratelli. We identify new potential donor genotypes for the genetic improvement of this character, and markers associated to QTLs that could be applied in rice breeding programs.

## Material and methods

### Phenotypic evaluation of rice varieties

A collection of 200 rice varieties adapted to temperate regions (S1 Table) was generated and seeds, harvested from plants cultivated in 2012 and 2013 under the conventional conditions used for commercial seed production in the East of Spain, were employed for germination experiments. Seeds were first surface sterilized with 1% NaClO (1/5 dilution of commercial bleach) for 15 min, and, after rinsed with sterile distilled H_2_O (three times), were transferred to plastic Petri dishes (9 cm diameter) containing two filter paper discs and 9 mL of sterile distilled H_2_O. Petri dishes covers were previously perforated in the centre (5 mm diameter) to avoid water condensation. For each variety at least three replicates with 20–25 seeds were prepared, and we used bulks of seeds produced in two years to reduce the effect of physiological differences. Seeds were then incubated in a growth chamber (CONVIRON ADAPTIS AR1000) with 70% HR and a photoperiod of 14 h of light, at 25° or 15°C. In the experiments performed at 25°C, seed germination and coleoptile length were monitored for 10 d or until rice seedlings reached 15 mm long, while rice seeds germinated at 15°C were monitored for 21 d. Four variables were employed to characterise rice seeds development under these suboptimal conditions: growth rate (coleoptile length/number of days) at 25°C (V25), growth rate at 15°C estimated after 21 d (V15), growth rate at 15°C estimated after the first 14 d of the trial (V1514d), and growth rate at 15°C during the third week of the trial (V153w), that is: (coleoptile length after 21 d—coleoptile length after 14 d)/7.

### Genotyping rice varieties

A panel of 2697 SNPs markers that covered the 12 rice chromosomes was developed as described by Reig-Valiente et al. [36]. This panel was employed to genotype the 200 rice varieties. After discarding heterozygous markers and those with missing data > 20% and with minor allele frequency (MAF) ≤ 5%, we obtained a set of 1672 SNPs for our association analyses.

### Genome association

Association analyses between SNP markers and seedling growth rates at 25 and 15°C were conducted using the software Tassel 5.2.23 [37] for the complete set of 200 rice varieties and for the *japonica* group of 180 cultivars (S1 Table). We first obtained a population structure matrix from a PCA with 11 components that explained 56% of total variance, using a panel of 944 unlinked markers, as derived from the previously estimated linkage disequilibrium [36]. For detecting significantly associated SNPs we tested three models: first, a general linear model containing only the SNPs as a fixed effect, with a matrix of population structure as covariate (GLM-PCA) and performing 10,000 replicates; second, a mixed linear model that includes a kinship matrix (MLM-K) as a random effect [38] and third, a MLM including both population structure and kinship relations (MLM-PCA-K). The MLM was used with two different kinship matrices, the Centered IBS matrix calculated in Tassel and that obtained from the kinship coefficients by [39], as implemented in the SPAGeDI software [40]. In this later matrix, all negative values between individuals were set to 0 [41]. The MLM analyses were conducted with the options of compression and re-evaluation of variances at each marker. We analysed the genotypes of the 200 or 180 cultivars for each set of markers associated with a trait with a *p*-value below 0.001. To corroborate the effects in each trait of the markers detected using different models, we estimated the Spearman correlation coefficient between the numbers of favourable alleles (as a SNP can be associated with a positive or negative effect) in each group of markers associated to a trait and the respective phenotypes for that trait. Finally, we estimated the proportion of significantly associated markers by applying a false discovery rate (FDR) procedure with *q* = 0.1 [42] and also performed permutation tests for each trait (*N* = 1000) using PLINK 1.9 Software [43] to set a critical threshold value [44]. This threshold *p* value was similar to that obtained with the modified Bonferroni threshold (*p* < 1/*n*, where n is the number of markers used for association mapping). The relative magnitude of each association was represented by the R^2^ value as the portion of variation explained by the marker.

## Results

### Germination of rice seeds at suboptimal temperatures

**Fig 1:**
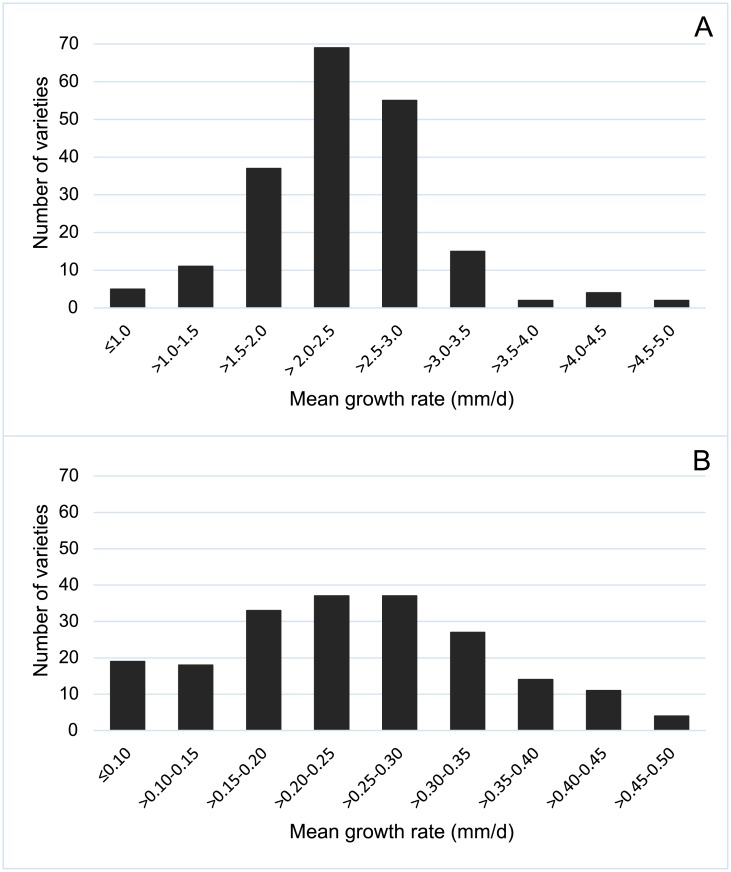
Variability for mean growth rate of coleoptiles (length/number of days) of 200 rice varieties when germinated at 25° (V25) until reached 15–20 mm long (A) and at 15°C (V15) for 21 days (B).

In our experimental conditions, most of the rice varieties (172 out of 200) reached percentages of germination higher than 90% when germinated at 25°C. In fact, more than 70% of the rice seeds germinated within two days. However, we found a wide continuous variation for mean coleoptile growth rate among varieties (Table 1), since V25 values ranged from 0.76 ± 0.15 mm/d of Labelle to 4.86 ± 0.78 mm/d of IR-64 seeds, with a mean of 2.38 ± 0.66 mm/d (Fig 1A). The faster development was observed in seeds of six varieties, two *indica* (IR-64 and IR-10198) and four *japonica* (Italica Livorno, Loto, Puita CL and Taichung-65), that showed coleoptile growth rates higher than 4.0 mm/d. As a result, after 10 days the percentage of seeds that produced viable seedlings (coleoptile length ≥ 6 mm) was higher than 90% for 160 varieties. In the opposite side, for two of the assayed varieties, Giglio and M-101, the percentage of viable seedlings produced was below 50%.

**Table 1:** Phenotypic variation for coleoptile growth rates (mm/d) during germination of rice seeds at 25° (V25) or 15°C (V15, V1514d, V153w).

Trait	200 cultivars				180 *japonica* cultivars			
	Mean	Range	CV^1^	K-S^2^	Mean	Range	CV^1^	K-S^2^
V25	2.38 ± 0.66	0.76–4.86	27.8%	NS	2.32 ± 0.63	0.76–4.07	25.3%	NS
V15	0.25 ± 0.10	0.02–0.50	40.6%	NS	0.25 ± 0.10	0.02–0.50	38.4%	NS
V1514d	0.17 ± 0.08	0.00–0.39	48.2%	<0.001	0.17 ± 0.08	0.00–0.39	48.1%	<0.001
V153w	0.41 ± 0.23	0.00–1.05	55.6%	0.011	0.42 ± 0.23	0.00–1.05	52.0%	NS

V25, coleoptile growth rate (mm/d) at 25°C; V15, coleoptile growth rate in 21 d at 15°C; V1514d, coleoptile growth rate in 14 d at 15°C; V153w, coleoptile growth rate in the 3^rd^ week of the trial at 15°C.

^1^Coefficient of variation

^2^*p*-value of the Kolmogorov-Smirnoff normality test.

**Fig 2:**
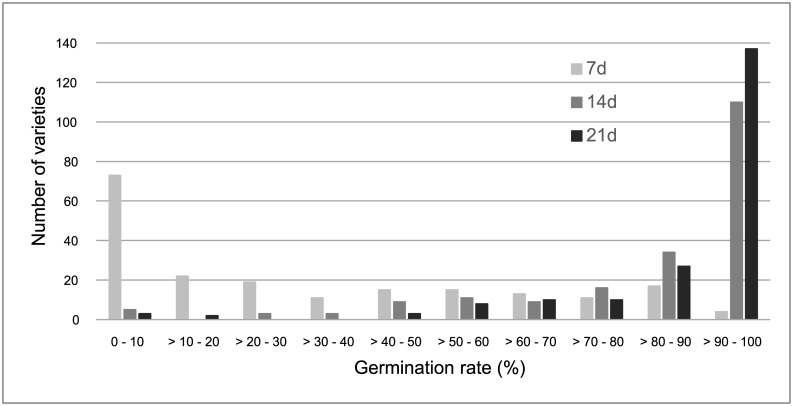
Variation in percentages of germinated seeds among 200 rice varieties assayed at 15°C after 7, 14 and 21 d.

**Fig 3:**
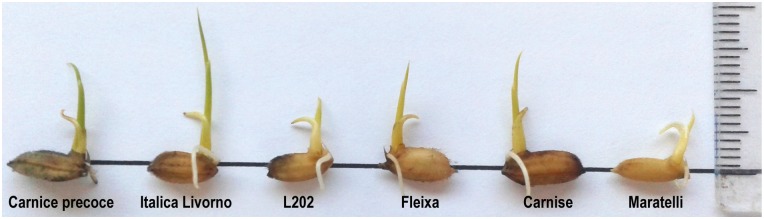
Variation in coleoptile length after 21 d at 15°C of germinating seeds of rice cultivars (from left to right) Carnice precoce, Italica Livorno, L-202, Fleixa, Carnise and Maratelli.

Coleoptile growth rates were substantially reduced when rice seeds were germinated at a lower temperature (15°C) (Table 1, Fig 1B) and we found again a wide variation among cultivars. For the complete trial, after 21 d, growth rates (V15) ranged from 0.02 ± 0.02 mm/d for seeds of Fragance to 0.50 ± 0.12 mm/d for Italica Livorno seeds, with a mean value of 0.25 ± 0.10 mm/d and with the highest rates showed by *japonica* varieties, as expected. Cold delayed germination of rice seeds, since most of them started germination within 5–7 days and coleoptile emergence continued along the assay (Fig 2) especially in the second week. Faster germination (more than 70% of germinated seeds after 7d) was observed for 32 varieties of different origins and types (e.g. Leda, Puita CL, Ripallo, Manuela, Loto, Ullal, Habataki, Savio). At the end of the experiment, 137 rice varieties showed percentages of germination higher than 90% at 15°C. In the opposite side, less of 20% of seeds from five varieties (Bluebelle, Giglio, Fragrance, Mangetsumochi and Agami) germinated after 21 d at 15°C. Although rice seeds could germinate under these conditions of low temperature, after the emergence of the coleoptile most of them stopped growing and did not elongate more than 3–5 mm. Furthermore, percentage of seeds producing viable seedlings after 14 d was less of 10% for 176 varieties. In contrast, seeds from some varieties maintained coleoptile growth rate during the three weeks of the assay, since 15 of them elongated at more than 0.4 mm/d. The higher growth rates at this limiting temperature were observed mostly in seeds of Italian varieties (Fig 3) as Italica Livorno, Carnise, Poseidone, Carnice precoce, Ducato, Savio, Ripallo, Nuovo Maratelli, and Karnak. Similar results were obtained for seeds of the Spanish varieties Fleixa, Gavina and Sivert, as well as for other varieties of different origins as S-101, Langhi and Akihikari.

When we compared the results obtained for the four variables, we found significant correlations between growth rate at 25°C (V25) and the initial growth rate at 15°C (V1514d), for both the complete set of varieties and for the *japonica* main group (Table 2). In these 180 cultivars we also estimated a low but significant correlation between growth rates at the two temperatures (Spearman coefficient 0.200, *p* < 0.001). Growth rate in cold conditions (V15) depended both on the initial germination rate (V1514d) and the coleoptile elongation within the third week of the trial (V153w), since significant correlations were estimated between these three parameters for the two sets of varieties (Table 2). Finally, we also observed a low correlation for *japonica* cultivars between the initial growth rate at 15°C and the growth rate in the third week of this experiment (0.200, *p* = 0.001), which could indicate the existence of different mechanisms of growth regulation, and then of cold tolerance, at the two stages of development of rice seedlings.

**Table 2:** Significant correlations (Spearman coefficients) among four coleoptile growth rates (mm/d) determined at 25 (V25) or 15°C (V15, V1514d, V153w).

Trait	V25	V15	V1514d	V153rdw
V25	1.000	0.200*	0.462**	
V15		1.000	0.697**	0.842**
V1514d	0.428**	0.688**	1.000	0.257*
V153w		0.833**	0.255**	1.000

V25, coleoptile growth rate (mm/d) at 25°C; V15, coleoptile growth rate in 21 d at 15°C; V1514d, coleoptile growth rate in 14 d at 15°C; V153w, coleoptile growth rate in the 3^rd^ week of the trial at 15°C.

Below diagonal, estimates for the complete set of 200 rice varieties; above diagonal, estimates for the *japonica* group (180 varieties).

*, significant *p* < 0.01;

**, significant *p* < 0.001.

### Association analyses

We initially performed association analyses based exclusively on the phenotype for the complete set of 200 rice cultivars and for the subset of 180 *japonica* varieties. We detected 451 and 194 significantly associated (*p* < 0.001) markers, respectively, but the number of detected markers was substantially reduced when population genetic structure, kinship relatedness, or both, were included in the analyses to control false associations (Table 3). Since the adoption of different levels of population structure and relatedness can effectively eliminate the excess of low *p*-values but it also likely eliminate true positives, we analysed all the SNPs detected for each trait under any model with a *p*-value < 0.001. The individual effects, positive and negative, of the detected markers on the phenotype were low (r^2^ = 0.067 on average), but contributed to explain the observed variability, since significant Spearman correlation coefficients were estimated for the number of favourable alleles in each panel of markers and the respective trait (data not shown). The genetic model also affected the significance of the detected marker-trait associations, especially for the complete data set and for PCA- based (GLM and MLM) or MLM-K models (S1 Fig). Most of the associated markers, however, were significantly detected in more than one approach, for example when using models with the two different kinship matrices. The effect of genetic structure on association analyses was different for the four traits: MLM-K detected more markers significantly associated to V25 than the two models that included PCA as a covariate, probably indicating an overestimated effect of population structure for this trait, for which the highest values were observed in both *indica* and *japonica* cultivars. In the analysis of the complete data set, two different groups of markers were detected whether the genetic structure was included or not as a covariate. The number of favourable alleles in the 7 markers detected by the GLM-PCA and the MLM-PCA-K models correlated with V25 values, as we estimated a significant Spearman coefficient of 0.152 (*p* = 0.032), while the Spearman correlation coefficient estimated between the number of favourable alleles in the 8 markers associated under the MLM-K model and V25 phenotypes was 0.555 (*p* < 0.001), and for the complete set of 14 markers (one SNP was detected in all models) the estimated Spearman coefficient was 0.475 (*p* < 0.001). The genetic structure based models were, then, less effective in detecting SNP markers associated to this trait. Similar results were observed for the markers associated to the correlated variable V1514d in the complete data set, since the Spearman coefficient estimated for the 7 markers detected by PCA-based models was 0.299 (*p* < 0.001), while the panel of 7 markers detected by the MLM-K analysis explained a higher percentage of variation (Spearman coefficient 0.435, *p* < 0.001) and the correlation coefficient between the number of favourable alleles in all 14 markers and V1514d phenotypes was 0.485 (*p* < 0.001). These results could be also due to the non-normal distribution of V1514d values.

**Table 3:** Number of markers, from the 1672 panel of SNPs, associated with the four analysed traits (*p* < 0.001) detected under three different models that control false associations (GLM-PCA, MLM-K and MLM-PCA+K), and percentages of associated markers referred to those detected in the phenotype analysis (GLM-Trait).

		GLM		MLM		
Data set	Trait	Trait	Trait+PCA	Trait+Kinship	Trait+PCA+Kinship	TOTAL
200	V25	174	2	8	7	14
	V15	123	3	1	4	5
	V1514d	33	7	7	1	14
	V153w	121	5	2	5	6
	*TOTAL*	*451*	*17*	*18*	*17*	*35*
	*%*	*27*.*0*	*3*.*8*	*4*.*0*	*3*.*8*	*7*.*8*
180	V25	60	2	4	3	7
	V15	37	5	2	3	8
	V1514d	28	13	7	3	13
	V153w	33	7	3	3	7
	*TOTAL*	*158*	*27*	*16*	*12*	*31*
	*%*	*9*.*4*	*17*.*1*	*10*.*1*	*7*.*6*	*19*.*6*

V25, coleoptile growth rate (mm/d) at 25°C; V15, coleoptile growth rate in 21 d at 15°C; V1514d, coleoptile growth rate in 14 d at 15°C; V153w, coleoptile growth rate in the 3^rd^ week of the trial at 15°C.

**Fig 4:**
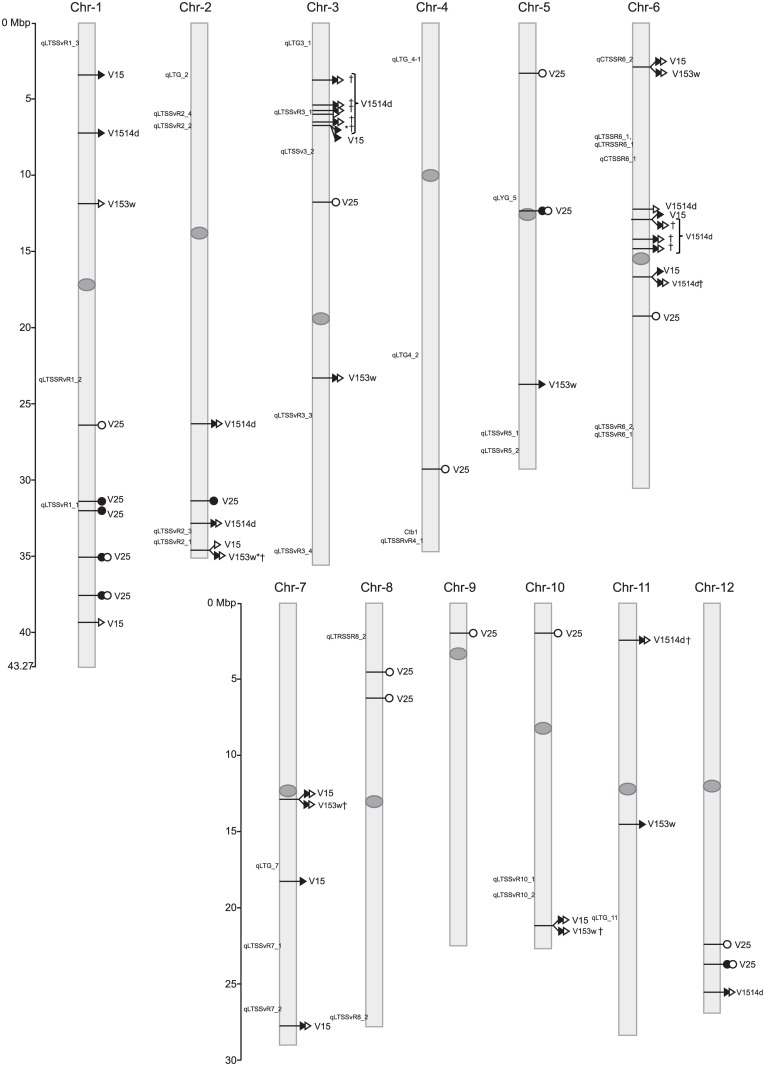
Chromosome position of the SNP markers associated with coleoptile growth rate of rice seeds germinated at 25°C (V25; ellipses) and to traits describing growth rate when seeds were germinated at 15°C (V15, V1514d and V153w; triangles). Association analyses were performed with the complete set of 200 rice varieties (white symbols) and with the 180 japonica cultivars (black symbols). †, markers with *p*-value < *q*-value, when *q*-value = 0.1*i*/1672; *, markers with *p*-values < 0.00005. In italics, previously described QTLs [27].

In total, 35 and 31 markers distributed in the 12 rice chromosomes were significantly (*p* < 0.001) detected in analyses performed for 200 and 180 cultivars, respectively. Some of them were significantly associated to more than one trait (Fig 4), as could be expected for these three parameters used to describe the variability observed in the development of rice seeds germinated at 15°C. However, we did not find any marker associated to growth rates at both 25° and 15°C temperatures in these data sets. Among the 31 markers identified in the 180 *japonica* rice cultivars, 7 were associated to V25 and 24 to V15, V1514d or V153w. When the two panels of detected SNPs were compared, we obtained a final set of 45 markers: 17 were associated to V25, and were distributed in 10 chromosomes (none found in chromosomes 7 and 11), while 11 SNPs were associated to V15 and, deepening into the genetic control of this trait, 16 markers were associated to V1514d and 8 to V153w. The 28 markers related to growth rates at 15°C were found in 9 chromosomes, since no associated markers were detected in chromosomes 4, 8 and 9. Among these 45 markers, 21 were detected in the analyses performed for the two data sets: 4 associated to V25 in chromosomes 1, 5 and 12, and 17 markers associated to V15, V1514d and/or V153w, which were distributed in chromosomes 2, 3, 6, 7, 10, 11 and 12. Furthermore, two wide regions of chromosomes 3 and 6, delimited by 4 and 5 SNP markers, respectively, were consistently associated to the ability of rice seedlings to grow at low temperature (Fig 4). Finally, to corroborate these results we combined the 31 markers associated to *japonica* phenotypes in a linear regression model that found that 27 loci significantly explained variation in V25 (r^2^ = 0.430, *p* < 0.001) and V15 (r^2^ = 0.531, *p* < 0.001). The remaining 4 markers are actually linked to some of these associated loci.

To clarify the effect of population structure on the genetic association for the V25 and V1514d traits, we performed a new association analysis in a subset of 173 cultivars that remained after removing *japonica* varieties (Bluebelle, Puita CL and Taichung-65, among others) assigned to the *indica* group by Bayesian-based genetic structure analyses previously reported [36]. In this data set, 9 SNP markers were associated to V25 values, four in chromosome 1 and five in chromosome 3, while 8 markers were associated to V1514d in chromosomes 2, 3 and 6. All markers had been detected in the previous analyses, being noticeable that three markers in chromosome 3 were associated to both traits. The numbers of favourable alleles in these two panels of markers correlated with the respective phenotypes, since the estimated Spearman coefficients were 0.335 (*p* < 0.001) with V25 and 0.477 (*p* < 0.001) with V1514d. This is in agreement to the correlation estimated among phenotypic values for both traits, and with the increasing values of V25 observed in *japonica* cultivars with higher number of favourable alleles for the 24 loci associated to V15, V1514d and V153w (Spearman coefficient 0.391, *p* < 0.001). On the contrary, *japonica* cultivars with higher number of favourable alleles for SNPs associated to V25 did not show better phenotypes for germination at 15°C, since we estimated no significant correlations. However, a low but significant correlation could be found between the number of favourable alleles for the 14 markers associated to V25 and the V1514d values (0.154, *p* = 0.030) for the complete set of 200 rice cultivars.

Finally, we selected a core panel of markers for the *japonica* group of varieties by applying a false discovery rate (FDR) method [42]. This method control spurious marker-trait associations that would still remain under GLM and MLM analyses, and allowed us to identify 13 markers distributed in six chromosomes (Fig 4) which showed the higher effects on growth rates at 15°C (Table 4). Two wide regions in chromosomes 3 and 6 were again tagged with 4 and 3 SNP markers, respectively. From our permutation analyses, in which GLM (marker-trait) association analyses were performed using 1000 permuted data sets for each trait, we estimated a mean critical threshold of 0.00005. Only two SNPs markers in chromosomes 2 and 3 satisfied this condition to declare significant association with a trait (Table 4).

**Table 4:** SNPs markers whith *p*-values that satisfied the FDR criterion with *q* = 0.1 for the *japonica* varieties group. The effect of each locus is estimated by the R^2^ value.

Trait	Chrom.	Position	R^2^
v153s	2	35406962	0.1004*
v1514d	3	3828648	0.0658
v1514d	3	5506710	0.0642
		5874917	0.0642
		6639548	0.0658
		6887497	0.0899*
v1514d	6	14483751	0.0673
		14612486	0.0651
		15152441	0.0597
v1514d	6	17024753	0.0688
v153s	7	13128278	0.0833
v153s	10	21628908	0.0831
v1514d	11	2502066	0.0815

V15, coleoptile growth rate in 21 d at 15°C; V1514d, coleoptile growth rate in 14 d at 15°C; V153w, coleoptile growth rate in the 3^rd^ week of the trial at 15°C.

*, markers with *p*-values < 0.00005.

### Selection of genotypes

**Fig 5:**
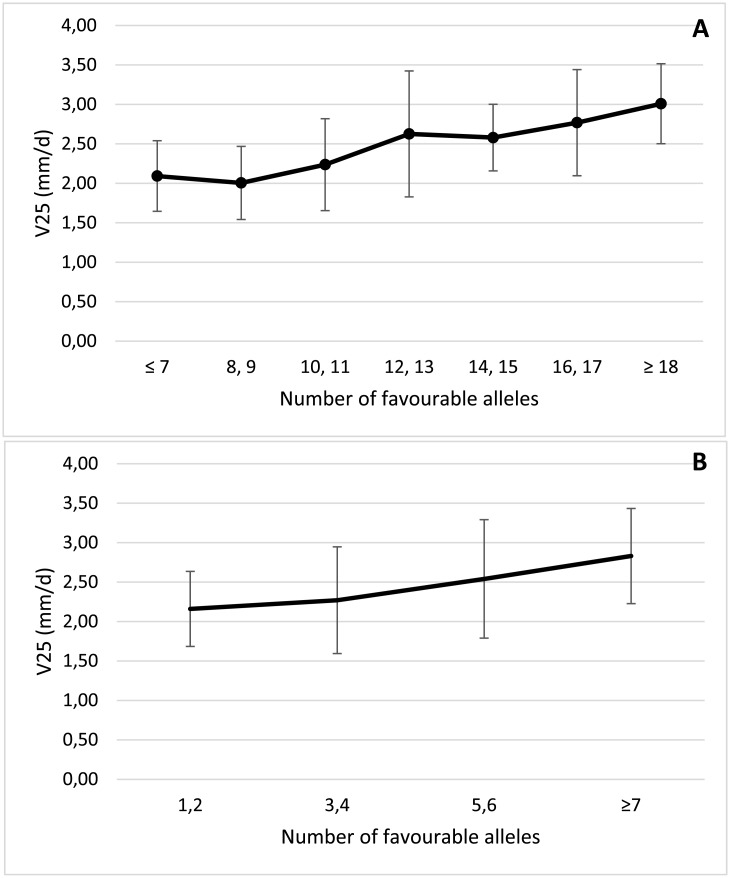
Mean and standard deviation for V25 of the 180 *japonica* rice cultivars with increasing number of favourable alleles in two panels of 31 (A) or 13 (B) SNP markers.

**Fig 6:**
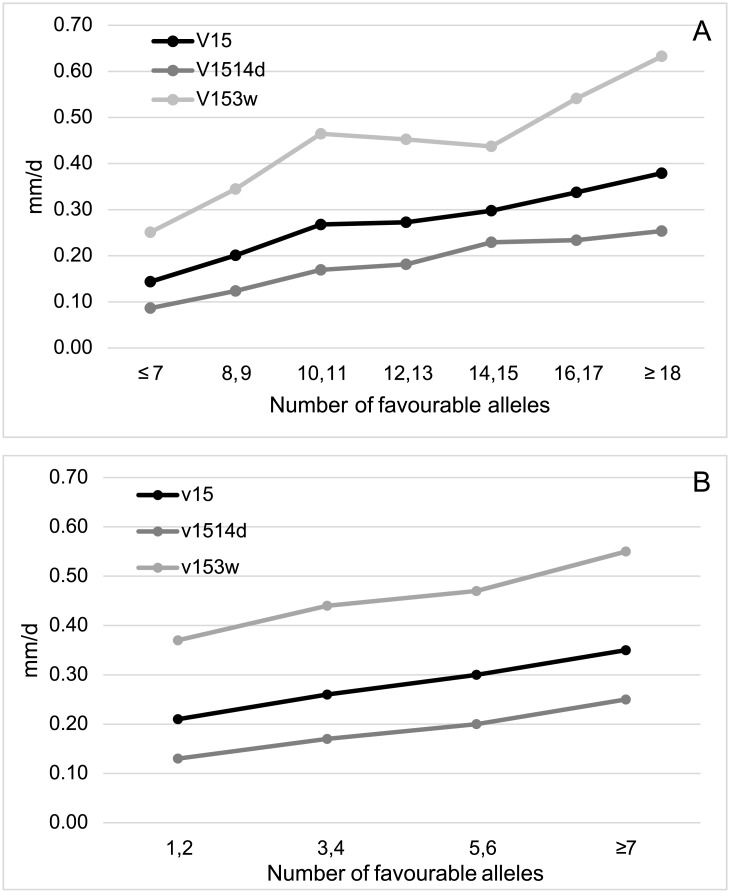
Mean values for V15, V1514d and V153w of the 180 *japonica* rice cultivars with increasing number of favourable alleles in two panels of 31 (A) or 13 (B) SNP markers.

We finally studied the genotypes of the 180 *japonica* cultivars for the two panels of 31 and 13 markers derived from our association analyses. Positive and significant Spearman correlation coefficients were estimated between the numbers of favourable alleles in each panel of markers and the phenotypes of these cultivars for the four traits (Table 5). These correlation coefficients were higher than those estimated between the numbers of favourable alleles in the panel of 35 markers detected in analyses for the complete data set of cultivars and the corresponding phenotypes. The effect of accumulating these alleles can be also observed when we averaged phenotypic values for V25 in *japonica* rice cultivars with increasing numbers of favourable alleles for the set of 31 (Fig 5A) or 13 (Fig 5B) associated markers. The same trend was obtained when we correlated the numbers of favourable alleles and the averaged values for V15, V1514d and V153w phenotypes, for both the 31 (Fig 6A) and the 13 SNP markers panels (Fig 6B). *Japonica* cultivars accumulating a higher number of favourable alleles for the panel of 31 SNP markers were mainly originated from Italy: Italica Livorno (22), Savio (20), Opale, Karnak, Giovani Marchetti, Carnise, Carnice precoce (19), Poseidone, Nuovo Maratelli and Genio (18). The cultivar Gema from Puerto Rico, which showed in our experiments superior but moderate growth rates when seeds were germinated at 15°C (0.34 mm/d) but low values for V25 (2.32 mm/d), also presented 18 favourable alleles. The same group of cultivars can be selected using data from the core panel of 13 markers, although the percentage of explained variation was lower (Table 5). Significant Spearman correlation coefficients (*p* < 0.001) were also estimated in the *japonica* varieties between the number of favourable alleles for the panel of 22 markers that are positive in the reference cultivar Italica Livorno and the values determined for V25 (0.348), V15 (0.597), V1514d (0.546) and V153w (0.438).

**Table 5:** Spearman correlation coefficients estimated between the phenotypes and the numbers of favourable alleles in three panels of markers (35, 13 and 31 SNPs) associated to four traits in the two sets of 200 and 180 rice cultivars.

	200	180 *japonica*	
Trait	35 markers	13 markers	31 markers
V25	0.443**	0.350**	0.494**
V15	0.310**	0.426**	0.554**
V1514d	0.378**	0.435**	0.577**
V153w	0.153*	0.232**	0.351**

V25, coleoptile growth rate (mm/d) at 25°C; V15, coleoptile growth rate in 21 d at 15°C; V1514d, coleoptile growth rate in 14 d at 15°C; V153w, coleoptile growth rate in the 3^rd^ week of the trial at 15°C.

*, significant *p* < 0.05;

**, significant *p* < 0.001.

## Discussion

Rice seeds from diverse cultivars, mostly *japonica* types adapted to temperate regions, showed a wide variation in germination and coleoptile elongation rates, and their ability for producing viable seedlings also depended greatly on temperature. On average, we estimated a reduction of 90% in rice seeds development rates when temperature was set from 25° to 15°C. Mean coleoptile growth rates were estimated using bulks of seeds produced in two years, to avoid a possible biass in phenotyping varieties due to variation in the physiological conditions of the seeds. We observed a differential performance of the 180 *japonica* cultivars under different temperatures, as revealed by the significant correlation coefficient estimated between V25 and V15 phenotypes (0.200, *p* = 0.007), and also a differential response during seed germination and coleoptile elongation, since the Spearman correlation coefficient between V1514d and V153w was also significant (0.257, *p* = 0.001). In a previous report, a GWAS of seed transcriptome at normal and low temperature revealed that the delayed development of rice seed in low temperature conditions is mainly caused by the inhibition of the development-related genes and that the transcriptional response of seed and seedling to low temperature is different [45]]. In our experiments, the highest mean growth rate when seeds were germinated at 15°C was determined for the cultivar Italica Livorno, for which Fujino et al. [19] referred a 100% germination rate after 7 days at this temperature. However, results for this reference cultivar did not significantly differ from those observed for other Italian cultivars as Carnice precoce, Carnise, Ducato, Poseidone or Savio, as well as for the American cultivar S-101, which could be then also employed as donor parents in breeding programs for improving this trait. The cultivars Maratelli and Nuovo Maratelli also showed relative high growth rates when germinated at 15°C (on average 0.38 ± 0.14 mm/d), but results were irregular in Maratelli seeds. In the opposite side, the Portuguese cultivar Arroz da Terra, which has also been reported to be cold tolerant at germination [30], did not show in our experiments a superior response. Although 86% of seeds from this cultivar germinated at 15°C, we determined a coleoptile mean growth rate of 0.26 ± 0.03 mm/d.

Using phenotypic data for the four variables and a panel of 1672 SNP markers, GLM and MLM-based association analyses identified markers significantly associated to these phenotypic traits, although allele effects were generally low. We detected markers associated to genomic regions with positive and negative effects on the analysed phenotypes. Significance of some marker associations varied when analyses were corrected with genetic structure as a covariate, especially for the complete data set of 200 cultivars, in which *japonica* types represented 90% of genotypes. Differences in model efficiency to detect false associations have been reported in association analyses performed in rice [46]. To avoid missing any potentially useful marker, we tested under any model the significance of the association of each panel of SNP markers detected for all traits by estimating the Spearman correlation coefficient between the number of favourable alleles in the panel of associated markers and the phenotypic value determined for each cultivar. We found then 31 SNP markers that explained variation in growth rates of *japonica* rice seeds germinated at suboptimal temperatures, and 21 of them were consistently detected when the non-*japonica* cultivars were also included in the analysis. Two different groups of markers were significantly associated to phenotypes determined in germination experiments performed at 25° and at 15°C. Some chromosome regions were associated to the ability to germinate at 15°C (V1514d) and others to the elongation of the coleoptile (V153w), all contributing to faster development of seeds at low temperature conditions. Similar results were referred by Jiang et al. [20] who detected eleven QTLs for LTG in a F_2_ population derived from the cross between USSR5 and N22, but the number of significant QTL and their magnitudes varied at the different germination times between 7 and 15 days.

As mentioned, several QTLs related to LTG (qLTG3-1, qLTG-3-2, qLTG-7, qLTG-9, qLTG-11 and qLTG-12) have been previously mapped in the rice genome [32–34]. Furthermore, qLTG-3-1 (position 219,712–220,923) was cloned and it was found that the expression of the located gene (Os03g0103300) is not induced by cold, but it is related to the promotion of seed germination, although its concrete function is not known [32]. In Gramene database (http://archive.gramene.org/qtl/) we also found five QTLs associated with low temperature germination (qLTG) that were mapped in chromosomes 2, 4, 5 and 11 in a F_2_ family derived from the cross Nipponbare x Kasalath [47]. The Japanese Q-TARO database (http://qtaro.abr.affrc.go.jp/qtab/table) describes two QTLs: qLTG-5 in the cultivar Milyang23 (position 10,787,462–18,944,887) and qLTG-7 in cultivar Kinmaze (position 17,530,626 to 22,912,990). The regions tagged in our analyses are located at different positions to the referred for these QTLs, most of them detected in mapping populations derived from *japonica* x *indica* crosses and using other traits to characterise low temperature tolerance, as germination rate or seedling survival rate. However, some QTL regions identified by Pan et al. [27] in chromosomes 1, 2, 3 and 6 are proximal to correlated SNPs herein found (Fig 4). Among the characterized genes located in these regions, according to the Rice Annotation Project (http://rice.plantbiology.msu.edu/index.shtml), we found enzymes involved in mechanisms of stress tolerance, such as thioredoxins (i.e. Os01g07376), which have been related to oxidative stress response, and leucine rich repeat proteins which mediate in disease resistance (i.e. Os12g37280). Furthermore, two gene products located at chromosomes 3 and 6, Os03g12820 and Os06g06400, were referred to be differentially expressed in rice seedlings subjected to different abiotic stresses [48]. Other product genes are enzymes that control development such as pentatricopeptide repeat domain containing proteins (Os02g57800, Os07g47470) and regulators of ribonucleases, as well as the scarecrow transcription factor (Os10g40390). Many targeted positions, particularly those in chromosomes 6 and 7, were associated to transposable elements (TEs), which is in interesting accordance with a study about the response of rice to virus infection [49]. TEs could account for up to 65% of the rice genome and their role in generating both new genes and alleles, and also in regulating gene expression, for example by providing cis-elements at promoter and enhancer regions, has been reviewed by Chénais et al. [50]. In this context, TEs also mediate in the response to stress and in the adaptation to environmental changes, since stress conditions induce in plants overexpression of TEs, hence generating genetic and epigenetic variability. New germination experiments are being performed in order to test if the expression of any of the tagged sequences, particularly the genes that co-localize with the two SNPs markers with stronger association (Os02g57800 and Os0312820) is induced by cold.

We finally investigated whether the combination of a higher number of favourable alleles in the panel of associated markers, considering both positive and negative effects as suggested by Pan et al. [27], correlated with increased phenotypic values. Irrespective of the analysed panel, significant Spearman correlation coefficients were estimated for the four phenotypic traits, which corroborate the utility of the detected markers for MAS for breeding *japonica* rice at LTG. To validate these panels, we have obtained several families of plants derived from crosses between deficient Spanish cultivars and donor varieties identified in this work, that will be used in future studies. We aim also to compare the effect of the introduction of these QTLs from different donor genotypes into different recipient genomes.

## Supporting information

S1 Fig Manhattan plots for the association between 1672 SNPs markers and coleoptile growth rates V25 (A, E), V15 (B, F), V1514d (C, G) and V153w (D, H) obtained for data of 200 rice varieties using the models GLM-PCA (A-D) or MLM (E-H) in TASSEL software. (PDF)

S1 Table Rice varieties from different origins and subspecies used in the association analyses. (DOCX)
