# Supplementary figures and images for: Genome wide association analysis of cold tolerance at germination in temperate japonica rice (Oryza sativa L.) varieties
Source: PLoS One. 2017 Aug 17;12(8):e0183416. doi: 10.1371/journal.pone.0183416 (PMC5560564; doi:10.1371/journal.pone.0183416)

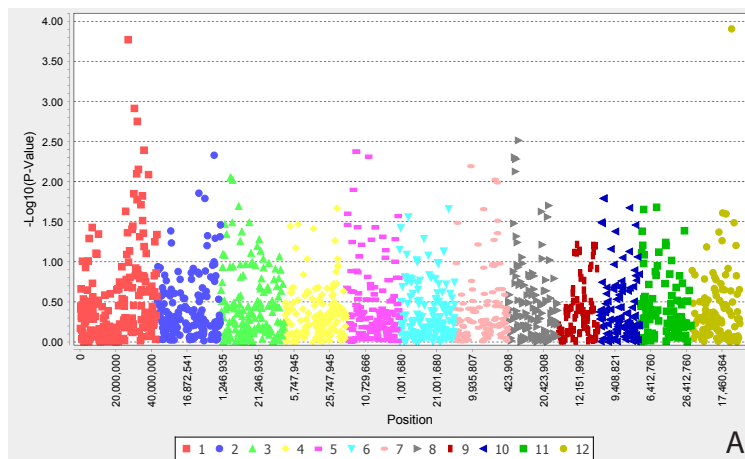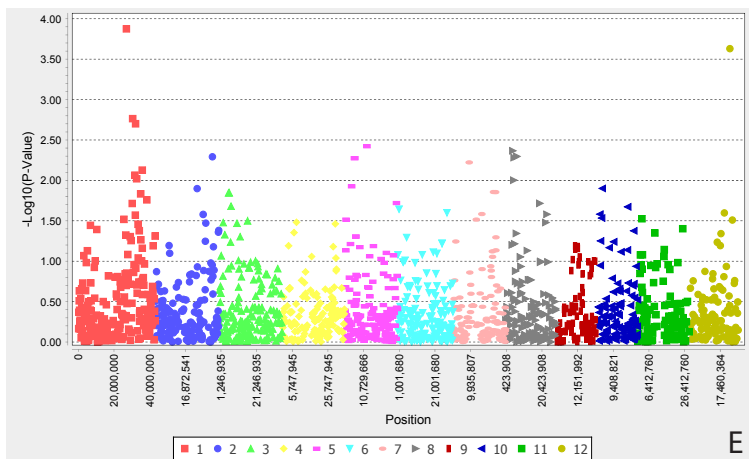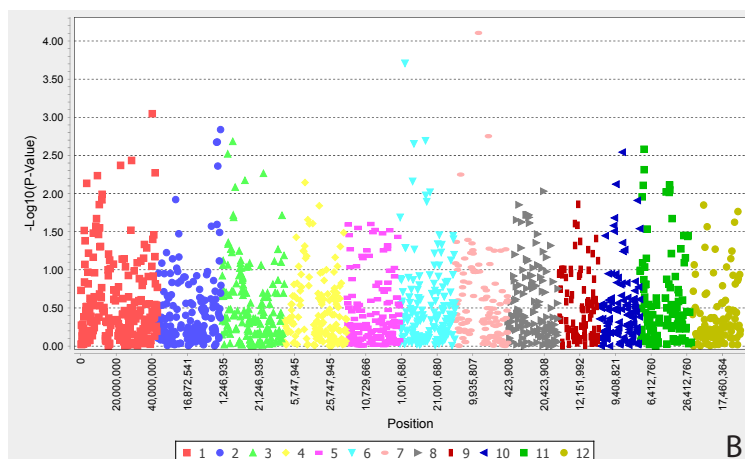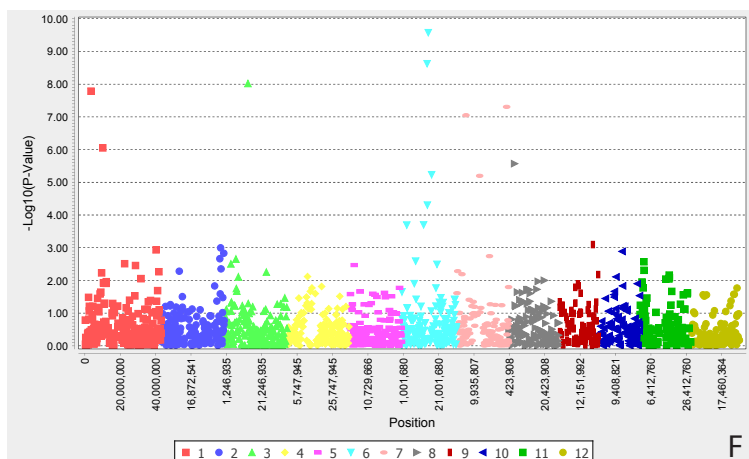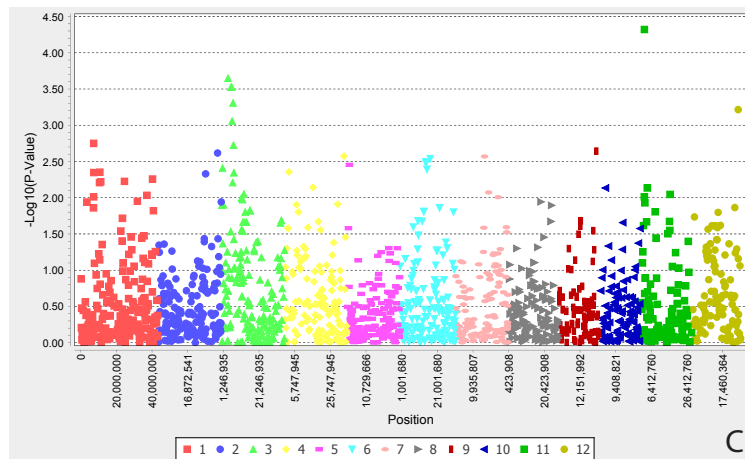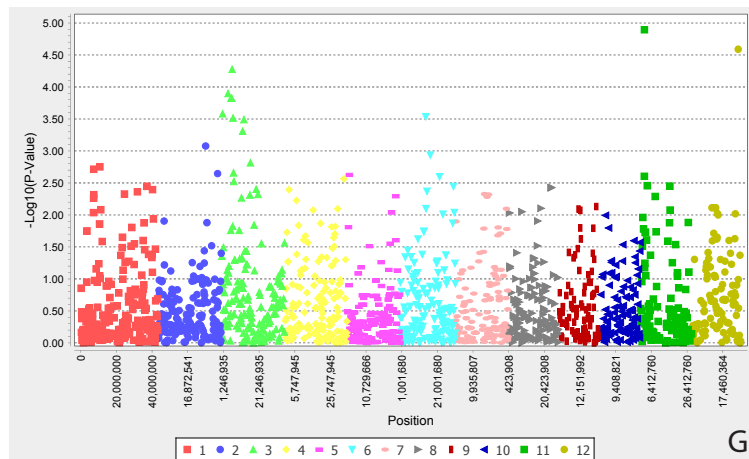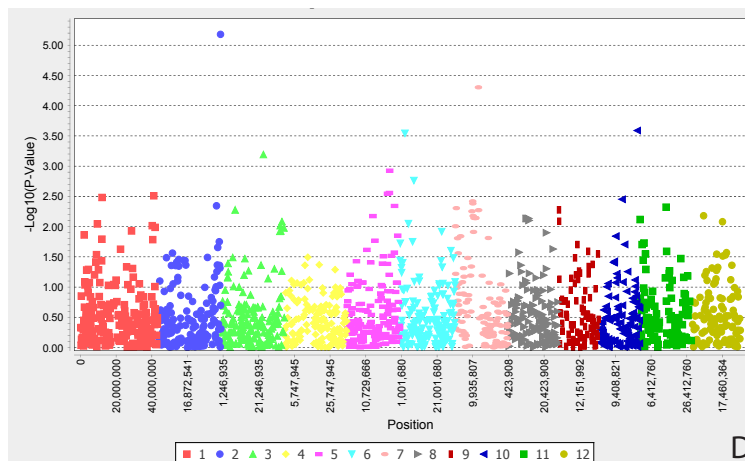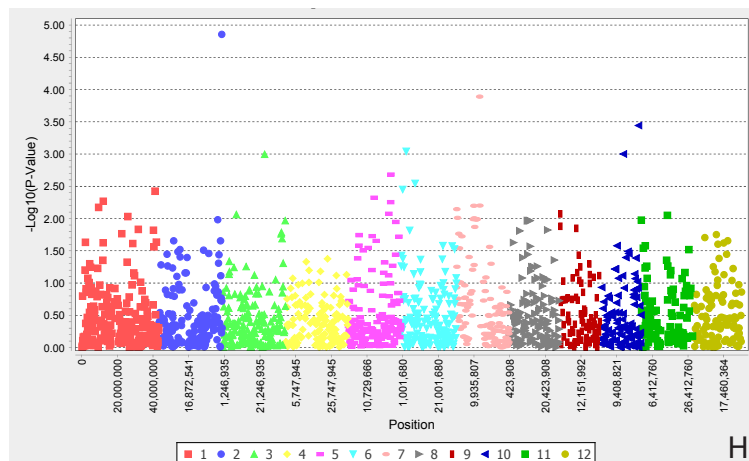

Supplement: S1 Fig — (PDF) [file pone.0183416.s001.pdf]
