# Supplementary material for: Genome wide association analysis of cold tolerance at germination in temperate japonica rice (Oryza sativa L.) varieties
Source: PLoS One. 2017 Aug 17;12(8):e0183416. doi: 10.1371/journal.pone.0183416 (PMC5560564; doi:10.1371/journal.pone.0183416)
Supplement: S1 Table — (DOCX) [file pone.0183416.s002.docx]

| **Variety** | **Origin** | **Subsp.** |  | **Variety** | **Origin** | **Subsp.** |
| --- | --- | --- | --- | --- | --- | --- |
| A-301 | USA | *japonica* |  | Irat 320 | French Guiana | admix |
| Adret | France | *japonica* |  | Irat 339 | French Guiana | admix |
| Adriano | France | *japonica* |  | Irat 350 | French Guiana | admix |
| Agami | Egypt | *japonica* |  | Irat 353 | French Guiana | admix |
| Akihikari | Korea | *japonica* |  | Italica Livorno | Italy | *japonica* |
| Albada | Spain | *japonica* |  | Jsendra | Spain | *japonica* |
| Albufera | Spain | *japonica* |  | Jucar | Spain | *japonica* |
| Alena | Spain | *japonica* |  | Kalao | France | *japonica* |
| Amaroo | Australia | *japonica* |  | Karnak | Italy | *japonica* |
| Antara | Spain | *japonica* |  | Kasalath | India | aus |
| Antares | Italy | *japonica* |  | Katy | USA | *japonica* |
| Apolo | Italy | *japonica* |  | Kitaake | Japan | *japonica* |
| Argila | Spain | *japonica* |  | Koshihikari | Japan | *japonica* |
| Arpa | Italy | *japonica* |  | Kyeema | Australia | *japonica* |
| Arroz da Terra | Portugal | *japonica* |  | L-202 | USA | *japonica* |
| Arsenal | Italy | *japonica* |  | L-203 | USA | *japonica* |
| Azucena | Philippines | *japonica* |  | L-206 | USA | *japonica* |
| Bahia | Spain | *japonica* |  | LA-110 | USA | *japonica* |
| Baixet | Spain | *japonica* |  | Labelle | USA | *japonica* |
| Balilla | Italy | *japonica* |  | Lady Wright | USA | *japonica* |
| Balilla x Sollana | Spain | *japonica* |  | Langhi | Australia | *japonica* |
| Bendret | Spain | *japonica* |  | Leda | Spain | *japonica* |
| Benisants | Spain | *japonica* |  | Lemont | USA | *japonica* |
| Benlloch | Spain | *japonica* |  | Lido | Italy | *japonica* |
| Betis | Spain | *japonica* |  | Liso | Spain | *japonica* |
| Bluebelle | USA | *japonica* |  | Litchikiang | China | *japonica* |
| Bomba | Spain | *japonica* |  | Llevant | Spain | *japonica* |
| Bombilla | Spain | *japonica* |  | Loto | Italy | *japonica* |
| Bombita | Spain | *japonica* |  | LTH | China | *japonica* |
| Bombon | Spain | *japonica* |  | M-101 | USA | *japonica* |
| Calbelle | USA | *japonica* |  | M-103 | USA | *japonica* |
| Calmati-202 | USA | *japonica* |  | M-104 | USA | *japonica* |
| Calmochi-101 | USA | *japonica* |  | M-201 | USA | *japonica* |
| Campino | Portugal | *japonica* |  | M-202 | USA | *japonica* |
| Capataz | Spain | *japonica* |  | M-203 | USA | *japonica* |
| Carmen | Spain | *japonica* |  | M-204 | USA | *japonica* |
| Carnice precoce | Italy | *japonica* |  | M-205 | USA | *japonica* |
| Carnise | Italy | *japonica* |  | M-206 | USA | *japonica* |
| Centa A-6 | El Salvador | *japonica* |  | M-208 | USA | *japonica* |
| Cerere | Italy | *japonica* |  | M-5 | USA | *japonica* |
| Cesare | Italy | *japonica* |  | M-9 | USA | *japonica* |
| Clavel | Spain | *japonica* |  | Mangetsumochi | Japan | *japonica* |
| Clot | Spain | *japonica* |  | Manuela | Spain | *japonica* |
| CNA-6159 | Brasil | ind x jap |  | Maratelli | Italy | *japonica* |
| Co39 | India | *indica* |  | Mareny | Spain | *japonica* |
| Colina | Spain | *japonica* |  | Marisma | Spain | *japonica* |
| Colusa | Spain | *japonica* |  | Marjal | Spain | *japonica* |
| Colusa x Nano | Spain | *japonica* |  | Mars | USA | *japonica* |
| Copsemar-7 | Spain | *japonica* |  | Matusaska | Japan | *japonica* |
| Cormorán | Spain | *japonica* |  | Maybelle | USA | *japonica* |
| CRLB1 | Italy | *japonica* |  | Millin | Australia | *japonica* |
| CT-13432 | Colombia | *indica* |  | Montsianell | Spain | *japonica* |
| CT-18 | Colombia | *japonica* |  | Nam Yang | Korea | *japonica* |
| Daegudo | Korea | *japonica* |  | Nano | Italy | *japonica* |
| Daekwanbyeo | Korea | *japonica* |  | Nano x Sollana | Spain | *japonica* |
| Daeseongbyeo | Korea | *japonica* |  | Newbonnet | USA | *japonica* |
| Dongjinbyeo | Korea | *japonica* |  | Nipponbare | Japan | *japonica* |
| Dosel | Spain | *japonica* |  | Niva | Spain | *japonica* |
| Drago | Italy | *japonica* |  | No 11 | Japan | *japonica* |
| Ducato | Italy | *japonica* |  | Norin 8 | Japan | *japonica* |
| Eridano | Italy | *japonica* |  | Northrose | USA | *japonica* |
| Fado | Spain | *japonica* |  | Nova 66 | USA | *japonica* |
| Fast | Italy | *japonica* |  | Nuovo Maratelli | Italy | *japonica* |
| Fedearroz-50 | Colombia | *indica* |  | Olesa | Spain | *japonica* |
| Fleixa | Spain | *japonica* |  | Opale | Italy | *japonica* |
| Fonsa | Spain | *japonica* |  | Oryzica Llanos 5 | Colombia | *indica* |
| Fragance | Italy | *japonica* |  | Pavlovsky | Russia | *japonica* |
| Frances | Spain | *japonica* |  | Pegonil | Spain | *japonica* |
| Fuzisaka | Japan | *japonica* |  | Piñana | Spain | *japonica* |
| Ganao | France | *japonica* |  | Poseidone | Italy | *japonica* |
| Gavina | Spain | *japonica* |  | Precoz-6 | Italy | *japonica* |
| Gema | Puerto Rico | *japonica* |  | Puita CL | Argentina | *japonica* |
| Genio | Italy | *japonica* |  | Puntal | Spain | *japonica* |
| Giano | Italy | *japonica* |  | Ricastello | Spain | *japonica* |
| Gigante Vercelli | Italy | *japonica* |  | Ripallo | Spain | *japonica* |
| Giglio | Italy | *japonica* |  | Rivera | Spain | *japonica* |
| Giovanni Marchetti | Italy | *japonica* |  | Romolo | Italy | *japonica* |
| Giza 178 | Egypt | *japonica* |  | S-101 | USA | *japonica* |
| Giza 181 | Egypt | *japonica* |  | Sarcet | Spain | *japonica* |
| Gladio | Italy | *indica* |  | SaT20 | Spain | *japonica* |
| Gleva | Spain | *japonica* |  | Saturn | USA | *japonica* |
| Goolarah | Australia | *japonica* |  | Savio | Italy | *japonica* |
| Guadiamar | Spain | *japonica* |  | Senia | Spain | *japonica* |
| Guweoldo | Korea | *japonica* |  | Sequial | Spain | *japonica* |
| Habataki | Malaysia | *indica* |  | Sigikari | Japan | *japonica* |
| Harra | Australia | *japonica* |  | SIS R215 | Italy | admix |
| Hidalgo | Spain | *japonica* |  | Sivert | Spain | *japonica* |
| Honduras | Honduras | *japonica* |  | Sole CL | Spain | *japonica* |
| Hwayoung | Korea | *japonica* |  | Sollana | Spain | *japonica* |
| Icaro | Italy | *japonica* |  | Susi | Spain | *japonica* |
| Ilang-Ilang | Korea | *japonica* |  | Taichung 65 | Taiwan | *japonica* |
| Illabong | Australia | *japonica* |  | Tainung 67 | Taiwan | *japonica* |
| Insen x Tremesino | Spain | *japonica* |  | Tebonnet | USA | *japonica* |
| IR-10198 | Philippines | *indica* |  | Tebre | Spain | *japonica* |
| IR-29 | Philippines | *indica* |  | Thaiperla | USA | *japonica* |
| IR-64 | Philippines | *indica* |  | Ullal | Spain | *japonica* |
| Irat 289 | French Guiana | admix |  | Urano | Italy | *japonica* |
| Irat 291 | French Guiana | admix |  | Venere | Italy | *japonica* |
| Irat 304 | French Guiana | admix |  | YR 3825-11-3-2-3-1 | Australia | *japonica* |
| Irat 307 | French Guiana | admix |  | YR 71011 | Australia | *japonica* |
